# Diaphragmatic Hernia in a Newborn With COL1A1-Associated Classical Ehlers–Danlos Syndrome

**DOI:** 10.1155/crig/5233998

**Published:** 2025-11-27

**Authors:** Laven Anand, Michael J. Munro, Ashalatha Shetty, John Dean, Judith Pagan, Jamie Campbell

**Affiliations:** ^1^School of Medicine, University of Dundee, Dundee, UK; ^2^Neonatal Unit, NHS Grampian, Aberdeen, UK; ^3^Royal College of Obstetricians and Gynaecologists, London, UK; ^4^Royal College of Physicians of Ireland, Dublin, Leinster, Ireland; ^5^School of Medicine Medical Sciences and Nutrition, University of Aberdeen, Aberdeen, UK; ^6^Department of Clinical Genetics, NHS Lothian, Edinburgh, UK

**Keywords:** classical Ehlers–Danlos syndrome, COL1A1, diaphragmatic hernia, newborn

## Abstract

Diaphragmatic rupture is an uncommonly seen complication of classical Ehlers–Danlos syndrome (cEDS). There have been no documented cases of diaphragmatic hernia in newborns having cEDS. This case study discusses a male infant delivered through spontaneous vertex delivery to a mother with cEDS. No evidence of a diaphragmatic hernia was found 6 days before delivery when an ultrasound scan to monitor a ventricular septal defect was carried out. Postnatally, the infant displayed signs of severe respiratory distress. A chest radiograph revealed a diaphragmatic hernia. The surgical team found and corrected a small posterolateral diaphragmatic defect on the third day of life. This resulted in a good recovery following management of a complication of chylothorax. The mother was known to have cEDS and bidirectional sequencing of the patient's lymphocyte DNA detected the heterozygous pathogenic familial variant *COL1A1* c.934C > T;p.(Arg312Cys). This variant has been previously reported in cases of cEDS. Other *COL1A1* variants are known to be associated with arthrochalasia-type EDS and osteogenesis imperfecta, but no *COL1A1* variants have been associated previously with congenital diaphragmatic hernia or diaphragmatic rupture. The familial variant impacts the highly conserved arginine residue in the Gly–X–Y triplet motif of the Type-I collagen protein. It has been reported in various families as a rare cause of autosomal-dominant cEDS. This case report details the patient's journey, including images of radiographs, highlighting a rare but important complication of spontaneous vertex delivery for individuals with cEDS. We also include a literature review on diaphragmatic hernia and rupture in classical EDS.

## 1. Introduction

**Figure 1:**
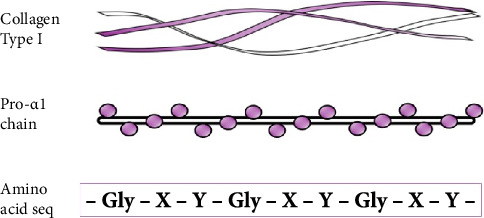
Illustration of the component parts of the Type I collagen. Top panel shows the triple helix of two pro-α1 chains and one pro-α2 chains. Middle panel shows a pro-α1 chain and the repeating motif. Bottom panel shows the amino acid sequence motif, critical for generating the triple helix.

Type I collagen is the most plentiful collagen subtype, found in cartilage, bone, tendon, skin and sclera. Type I collagen has a triple helical structure composed of two pro-α1 chains, encoded by the gene *COL1A1* and one pro-α2 chains encoded by *COL1A2*. Critical to the triple helix is a repeating amino acid triplet “Gly–X–Y,” and disruption of this motif is an established mechanism of the disease in connective tissue disorders (Figure 1). Pathogenic variants in *COL1A1* have a strong genotype-to-phenotype correlation. Pathogenic variants in glycine residues are commonly associated with osteogenesis imperfecta [1], and the p.(Arg1014Cys) substitution and p.(Arg918Cys) substitution are known to cause Caffey disease [2]. Deletion of exon 6 causes arthrochalasia type-EDS [3]. The variant p.(Arg312Cys) in COL1A1 is a rare cause of classical-type Ehlers–Danlos syndrome (cEDS) and is reported to have an increased association with vascular complications in some families [4–6]. Mutations in COL1A1 and COL1A2 have also been linked to cases of osteogenesis imperfecta/Ehlers–Danlos overlap syndrome, an allelic disorder which is characterised by patients displaying mixed phenotype of both conditions, which can include joint laxity, atrophic scars, easy bruising, bone abnormality, blue sclerae and vascular fragility [7, 8]. Vascular fragility is uncommon but an important and recognised feature of OIEDS overlap syndrome; cases include carotid artery dissection, carotid artery aneurysm and paraspinal vascular malformation [7].

Congenital diaphragmatic hernia (CDH) refers to the herniation of foetal abdominal organs into the thorax due to an opening in the diaphragm present at or before birth. This is due to incomplete closure of the diaphragm during foetal development. It is important to recognise the difference between CDH and spontaneous diaphragmatic rupture. While CDH is present at birth, spontaneous diaphragmatic rupture develops after birth due to weakening of the diaphragm or trauma.

The incidence of CDH varies globally and can range from 1 in 2000 to 1 in 5000 [9]. CDH is believed to have a multifactorial cause including genetics, environment and/or nutrition. Genetic causes can range from single-gene mutations to chromosomal abnormalities such as aneuploidy. It is well known that CDH is strongly linked to many aneuploidies including trisomy 21, trisomy 18 and trisomy 13.

cEDS is characterised by hyperextensible, fragile skin, easy bruising, hypermobile joints and atrophic scarring [4]. It is most commonly caused by pathogenic variants of *COL5A1* and *COL5A2*, both of which code for Type V collagen, but it can also be rarely caused by variants of *COL1A1* which codes for Type I collagen.

## 2. Results

### 2.1. Clinical Presentation and Family History

The proband in this family is a 31-year-old Caucasian female, clinically diagnosed with cEDS as a child. Molecular testing detected the heterozygous *COL1A1* variant c.934C > T p.(Arg312Cys), confirming the diagnosis. The patient had been made aware postdiagnosis that prenatal testing was available for any pregnancies in future. This was not pursued by her when she did get pregnant with the subject discussed in this case. She had an uneventful second pregnancy except for additional scans in the final trimester due to the detection of a small muscular ventricular septal defect (VSD) at a detailed scan at 20 weeks and for growth monitoring. An ultrasound scan of the foetus 6 days prior to delivery showed no overt signs of pathology within the chest.

**Figure 2:**
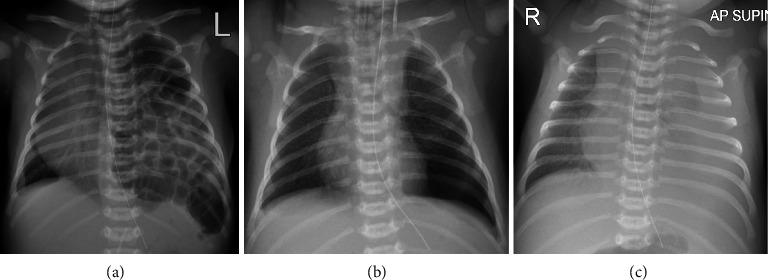
Chest radiographs for the subject. (a) Preoperative film with bowel loops in the left hemithorax. (b) Postoperative with normal appearance. (c) “Whiteout” of the left hemithorax at Day 10 of life caused by chylothorax.

The subject of this report is a Caucasian male born at 41 + 3 weeks' gestation by spontaneous vertex delivery. Delivery was precipitant with Apgar scores of 2, 7 and 9 at 1, 5 and 10 min, respectively. There were no wound healing issues in the mother noted in this case, likely due to spontaneous vaginal birth. Respiratory distress was noted after delivery, and the patient was moved to the neonatal unit with CPAP support commenced. A chest radiograph showed bowel loops in the left side of the thorax, with displacement of the heart and mediastinum to the right (Figures 2(a), 2(b), 2(c)). A diagnosis of diaphragmatic hernia was made, and the baby was sedated, intubated and ventilated. Surgical repair was performed by abdominal approach on Day 3 of life. Direct visualisation revealed a Type B diaphragmatic hernia due to a small posterolateral defect in the left side of the diaphragm. Recovery from surgery was complicated by chylothorax diagnosed on Day 10 of life, after establishment of full enteral feeds. The chylothorax was treated with enteral formula feeding with reduced long-chain triglyceride content, with good effect. He required 9 days of ventilation and seven further days of high-flow nasal cannula with supplemental oxygen. Examination revealed a transverse scar at the left subcostal region, hyperextensible skin with some redundancy of the skin at the dorsum of the hands and a minor umbilical hernia. The antenatally detected VSD was clinically undetectable. On Day 7 of life, growth parameters were unremarkable; weight 3770 g (Z + 0.113), length 50 cm (Z − 0.78) and head circumference 36 cm (Z + 0.547). No issues with wound healing were observed in the infant over the course of this case. Genetic testing in the neonatal period confirmed inheritance of the maternal variant in *COL1A1* compatible with a diagnosis of cEDS in this infant.

### 2.2. Genomic Analyses

• The NM_000088.4(COL1A1):c.934C > T p.(Arg312Cys) variant alters a highly evolutionarily conserved amino acid residue at position X within the collagen triple helix domain (Gly–X–Y repeat). It is absent from the gnomAD database (gnomAD v4.1.0). The variant is well documented in the literature, showing segregation with disease in families with one case reported as a de novo event [10]. Functional studies confirm this variant disturbs fibrillogenesis [3]. Therefore, this variant could be classified as pathogenic by Association for Clinical Genomic Science Guidelines [11], meeting criteria PP1_strong, PM2, PM6, PP4_moderate, PP2, PP3 and PS3_supporting.

## 3. Discussion

Diaphragmatic hernia/rupture has been reported in association with EDS, but this is most often the vascular subtype [9]. This case is notable for the normal scans closely preceding delivery. This could suggest an acquired diaphragmatic rupture, during labour, delivery or resuscitation rather than a primary defect of diaphragmatic development. Consideration should be given to whether the management of labour can be optimised to avoid precipitant delivery or a prolonged second stage for infants with cEDS or those at significant risk of having this condition. There are various reported pregnancy-related risks when both the mother and foetus have cEDS. These include perineal tearing, vaginal tearing, postpartum haemorrhage, preterm premature rupture of membranes and premature delivery. As there are no uniform guidelines to manage pregnancy and delivery in such patients, birth management must be considered on a case-by-case basis. The benefit of caesarean section delivery, to avoid compressive force in labour, needs to be weighed against the risks of complications from surgical intervention for women who are known to be affected by cEDS.

This is the first report of *COL1A1* cEDS associated with diaphragmatic hernia in a newborn. There are previous reports of CDH with osteogenesis imperfecta, but this likely represents a different underlying disease mechanism [8]. Previous case series and reports suggest an increased risk of vascular complications associated with this variant in *COL1A1*, but there is incomplete penetrance [5]. Thus, while reports of cardiovascular events with this *COL1A1* variant are uncommon, some patients with the variant have been reported to have cardiac and/or vascular complications such as mitral regurgitation, aortic regurgitation, vertebral artery tortuosity, iliac artery aneurysm and dissection and coeliac trunk aneurysm and dissection, usually in adult life [12]. It is hypothesised that there is a genotype–environment interaction leading to these vascular complications. Thus, lifestyle factors, such as proactive management of weight and diet and avoidance of smoking and high blood pressure, are important in this patient group. There is a lack of evidence about whether cardiovascular screening might be useful in patients with *COL1A1* c.934C > T p.(Arg312Cys) variant, but regular echocardiograms through childhood and adulthood are a possible method for screening and monitoring such patients. The family in this report was followed up with 3–5 yearly ultrasound scans in adulthood and imaging the thoracic and abdominal aorta in secondary care, as well as an annual review of blood pressure with their general practitioner.

## Data Availability

The authors have nothing to report.

## Supporting Information

CARE checklist of information which lists the information that has been provided in the case report submission, along with its exact location by line number.
